# Long thrombus with a removed microaxial blood pump catheter 10 days after ECPELLA support

**DOI:** 10.1093/ehjcr/ytad538

**Published:** 2023-11-02

**Authors:** Kazuyoshi Takagi, Kosuke Saku, Eiki Tayama

**Affiliations:** Division of Cardiovascular Surgery, Department of Surgery, Kurume University School of Medicine, 67 Asahimachi, Kurume 830-0011, Japan; Division of Cardiovascular Surgery, Department of Surgery, Kurume University School of Medicine, 67 Asahimachi, Kurume 830-0011, Japan; Division of Cardiovascular Surgery, Department of Surgery, Kurume University School of Medicine, 67 Asahimachi, Kurume 830-0011, Japan

## Case description

**Figure 1:**
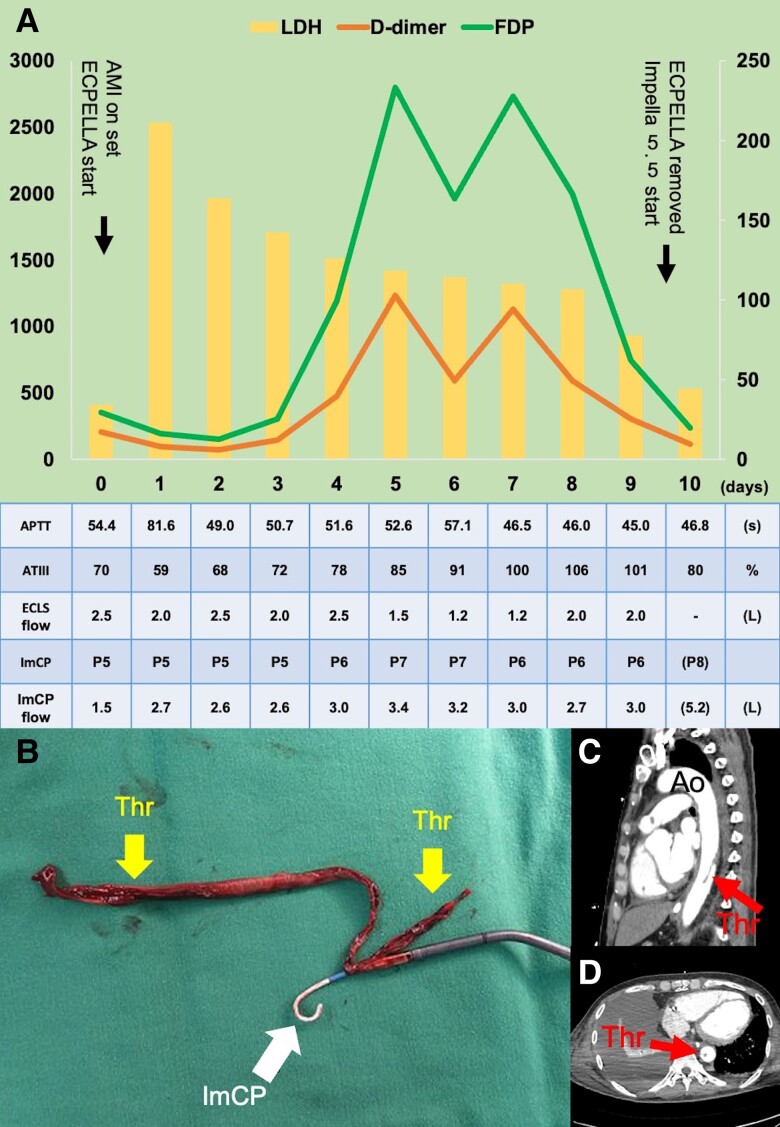
Time course of the levels of D-dimer (μg/mL), fibrinogen degradation products (μg/mL), lactate dehydrogenase (U/L), specific laboratory parameters to monitor the anticoagulation therapy, and the performance level of mechanical circulatory assist. The level of lactate dehydrogenase is on the left axis, and the levels of D-dimer and fibrinogen degradation products are on the right axis (*A*). Long thrombus with removed Impella CP SmartAssist 10 days after a transfemoral implantation (*B*) and residual thrombus formation in the descending aorta (*C* and *D*). LDH, lactate dehydrogenase; FDP, fibrinogen degradation products; AMI, acute myocardial infarction; APTT, activated partial thromboplastin time; ATIII, antithrombin levels; ECLS, extracorporeal life support; ImCP, Impella CP SmartAssist; Thr, thrombus; Ao, descending aorta.

A 61-year-old male with Type 2 diabetes was introduced to ECPELLA combined with a transfemoral Impella CP SmartAssist (ImCP) and extracorporeal life support (ECLS) following a 40 min cardiac arrest due to acute myocardial infarction (AMI). Our standard ECLS anticoagulation protocol targets an activated clotting time (ACT) of 180 s or higher; however, in this case, it was adjusted to a range of 160–180 s to prevent Impella-related bleeding complications. Four days after ECPELLA initiation, there were notable increases in D-dimer and fibrinogen degradation products (FDPs) despite appropriate anticoagulation therapy, particularly following a reduction in ECLS flow. Additionally, there was a sustained high level of lactate dehydrogenase (LDH) during ECPELLA use (*Figure 1A*). Impella CP SmartAssist was upgraded to Impella 5.5 with SmartAssist, and ECLS was discontinued after 10 days to provide extended mechanical circulatory support. Although ImCP functioned normally (*Figure 1A*) and echocardiography showed no visible thrombus, a 20 cm thrombus was found during ImCP removal (*Figure 1B*). Postoperative computed tomography (CT) scans revealed residual thrombus in the descending aorta (*Figure 1C* and *D*). The removal of ImCP with the thrombus led to significant decreases in D-dimer, FDP, and LDH levels. Anticoagulation therapy with an ACT exceeding 180 s was maintained. The patient received a durable ventricular assist device (VAD) 49 days after Impella 5.5 implantation, with no complications related to residual thrombus.

Thrombus formation associated with ImCP is rare,^1,2^ and reports of long thrombi post-removal are scarce. While ECPELLA shows promise in improving outcomes for cardiogenic shock due to prophylactic ventricular unloading,^3^ a case of visceral malperfusion has been reported.^2^ Possible explanations for this complication include enhanced thrombus formation in the descending aorta due to competitive flow between ImCP and ECLS, as well as adhered thrombus at the pigtail site or shaft of ImCP.^2^ Our case highlights that the flow balance between Impella and ECLS may be a factor in promoting thrombus formation. Confirming anticoagulation markers, an enhanced anticoagulation regimen according to these markers, and early withdrawal from ECPELLA in favour of transitioning to a VAD may reduce the risk of these complications. Performing enhanced CT imaging is recommended and proves valuable in detecting thrombus formation, which is often undetected by routine echocardiography, especially if thrombus formation is suspected. Clinicians should remain vigilant regarding the risk of thrombo-embolic complications during support and device explantation.

## Data Availability

The data underlying this article are available in the article.

## Notes

**IRB number**: Kurume University IRB No.21001, dated 20 April 2021.

**Consent**: Written informed consent was obtained for this report.

**Funding:** None declared.
